# COVID-19 Clinical Characteristics, and Sex-Specific Risk of Mortality: Systematic Review and Meta-Analysis

**DOI:** 10.3389/fmed.2020.00459

**Published:** 2020-07-21

**Authors:** Mohammad Javad Nasiri, Sara Haddadi, Azin Tahvildari, Yeganeh Farsi, Mahta Arbabi, Saba Hasanzadeh, Parnian Jamshidi, Mukunthan Murthi, Mehdi Mirsaeidi

**Affiliations:** ^1^Department of Microbiology, School of Medicine, Shahid Beheshti University of Medical Sciences, Tehran, Iran; ^2^Division of Pulmonary and Critical Care, Department of Medicine, University of Miami Miller School of Medicine, Miami, FL, United States; ^3^Student Research Committee, School of Medicine, Shahid Beheshti University of Medical Sciences, Tehran, Iran; ^4^Department of Pulmonary and Critical Care, Miami VA Medical Center, Miami, FL, United States

**Keywords:** coronavirus, COVID-19, mortality, male, pandemic

## Abstract

**Background:** The rapidly evolving coronavirus disease 2019 (COVID-19), was declared a pandemic by the World Health Organization on March 11, 2020. It was first detected in the Wuhan city of China and has spread globally resulting in a substantial health and economic crisis in many countries. Observational studies have partially identified different aspects of this disease. There have been no published systematic reviews that combine clinical, laboratory, epidemiologic, and mortality findings. Also, the effect of gender on the outcomes of COVID-19 has not been well-defined.

**Methods:** We reviewed the scientific literature published from January 1, 2019 to May 29, 2020. Statistical analyses were performed with STATA (version 14, IC; Stata Corporation, College Station, TX, USA). The pooled frequency with 95% confidence intervals (CI) was assessed using random effect model. *P* < 0.05 was considered a statistically significant publication bias.

**Results:** Out of 1,223 studies, 34 satisfied the inclusion criteria. A total of 5,057 patients with a mean age of 49 years were evaluated. Fever (83.0%, CI 77.5–87.6) and cough (65.2%, CI 58.6–71.2) were the most common symptoms. The most prevalent comorbidities were hypertension (18.5%, CI 12.7–24.4) and Cardiovascular disease (14.9%, CI 6.0–23.8). Among the laboratory abnormalities, elevated C-Reactive Protein (CRP) (72.0%, CI 54.3–84.6) and lymphopenia (50.1%, CI 38.0–62.4) were the most common. Bilateral ground-glass opacities (66.0%, CI 51.1–78.0) was the most common CT scan presentation. The pooled mortality rate was 6.6%, with males having significantly higher mortality compared to females (OR 3.4; 95% CI 1.2–9.1, *P* = 0.01).

**Conclusion:** COVID-19 has caused a significant number of hospitalization and mortality worldwide. Mortality associated with COVID-19 was higher in our study compared to the previous reports from China. The mortality was significantly higher among the hospitalized male group. Further studies are required to evaluate the effect of different variables resulting in sex disparity in COVID-19 mortality.

## Introduction

Facing an immediate crisis by the novel coronavirus, Severe Acute Respiratory Syndrome Coronavirus 2 (SARS-CoV-2) which has been called the once in a century pathogen, requires a global response (1). The disease caused by this virus has been named “coronavirus disease 2019” (COVID-19) by the World Health Organization (WHO). As of now, more than 180 countries have reported COVID-19 patients. Given the increasing number of countries infected with SARS-CoV-2, WHO finally classified COVID-19 as a pandemic on March 11, 2020 (2). The SARS-CoV-2 virus is a beta-coronavirus, belonging to the same coronavirus family as the Middle East Respiratory Syndrome virus (MERS-CoV) and SARS-CoV. MERS-CoV and SARS-CoV were previously responsible for respiratory syndrome outbreaks. However, COVID-19 is the first virus of the coronavirus family to cause a pandemic (3).

COVID-19 started in China in December 2019 when a cluster of patients with pneumonia of unknown origin were identified in the city of Wuhan. Since then, it has infected hundreds of thousands of people around the world and resulted in more than 539,900 deaths up to this date (4). Despite governmental travel restrictions in many countries, the confirmed number of new cases has been rising globally. The international community has asked for at least 675 million US dollars to use for preparedness and protection of states with weaker health systems (5).

In the previous two outbreaks of coronaviral respiratory illness, namely Severe Acute Respiratory Illness (SARS) and Middle East Respiratory Illness (MERS), gender-based differences in mortality were observed. In SARS, mortality risk was twice as high in younger males compared to younger females, but this difference in mortality decreased with older age. Additionally, the case fatality rate observed in males was twice that of females in MERS (6). The effect of sex on COVID-19 mortality was unknown. In our systematic review, we compared male and female mortality risk for COVID-19.

The novelty of COVID-19 has raised many questions about the epidemiology of the disease, clinical and laboratory methods of diagnosis, as well as therapeutic measures. Many observational studies have been dealing with these features separately. Further combined systematic reviews are needed, to understand the role of sex in COVID-19 associated mortality. In this meta-analysis study, we reviewed the published literature from January 1, 2019 to May 29, 2020 to provide a comprehensive overview of COVID-19.

## Methods

### Search Strategy

We searched Pubmed/Medline, Embase, Web of Science, and the Cochrane Library for studies published from January 1, 2019 to May 29, 2020. The search strategy was based on the following key-words: COVID-19, severe acute respiratory syndrome coronavirus 2, novel coronavirus, SARS-CoV-2, nCoV disease, SARS2, COVID19, Wuhan coronavirus, Wuhan seafood market pneumonia virus, 2019-nCoV, coronavirus disease-19, coronavirus disease 2019, 2019 novel coronavirus and Wuhan pneumonia. Lists of references of selected articles and relevant review articles were hand-searched to identify further studies. This study was conducted and reported according to the PRISMA guidelines (7). The study did not require Institutional Review Board approval.

### Study Selection

The records found through database searching were merged and the duplicates were removed using EndNote X7 (Thomson Reuters, New York, NY, USA). Two reviewers (YF and PJ) independently screened the records by title and abstract to exclude those not related to the current study. The full texts of potentially eligible records were retrieved and evaluated by a third reviewer (AT). Included studies met the following inclusion criteria: (i) patients were confirmed and diagnosed with RT-PCR as suggested by WHO; (ii) The raw data for clinical, radiological and laboratory findings were included; and (iii) the outcomes were addressed. Studies with insufficient information about patients' characteristics and outcomes were excluded. Case reports, reviews, and animal studies were also excluded. Only studies written in English were selected.

### Data Extraction and Quality Assessment

A data extraction form was designed by two reviewers (AZ and SH). These reviewers extracted the data from all eligible studies and differences were resolved by consensus. The following data was extracted: first author name; year of publication; type of study; country(ies) where the study was conducted; distribution of age and sex in the population; number of patients investigated; data for clinical, radiological, and laboratory findings; and outcomes.

### Data Synthesis and Analysis

Statistical analyses were performed with STATA (version 14, IC; Stata Corporation, College Station, TX, USA). The pooled frequency with 95% confidence intervals (CI) was assessed using random effect model. The between-study heterogeneity was assessed by Cochran's Q and the I2 statistic. Publication bias was assessed statistically by using Begg's and Egger's tests (*p* < 0.05 was considered indicative of statistically significant publication bias).

### Quality Assessment

The checklist provided by the Joanna Briggs Institute (JBI) was used to perform quality assessment (8).

## Results

**Figure 1:**
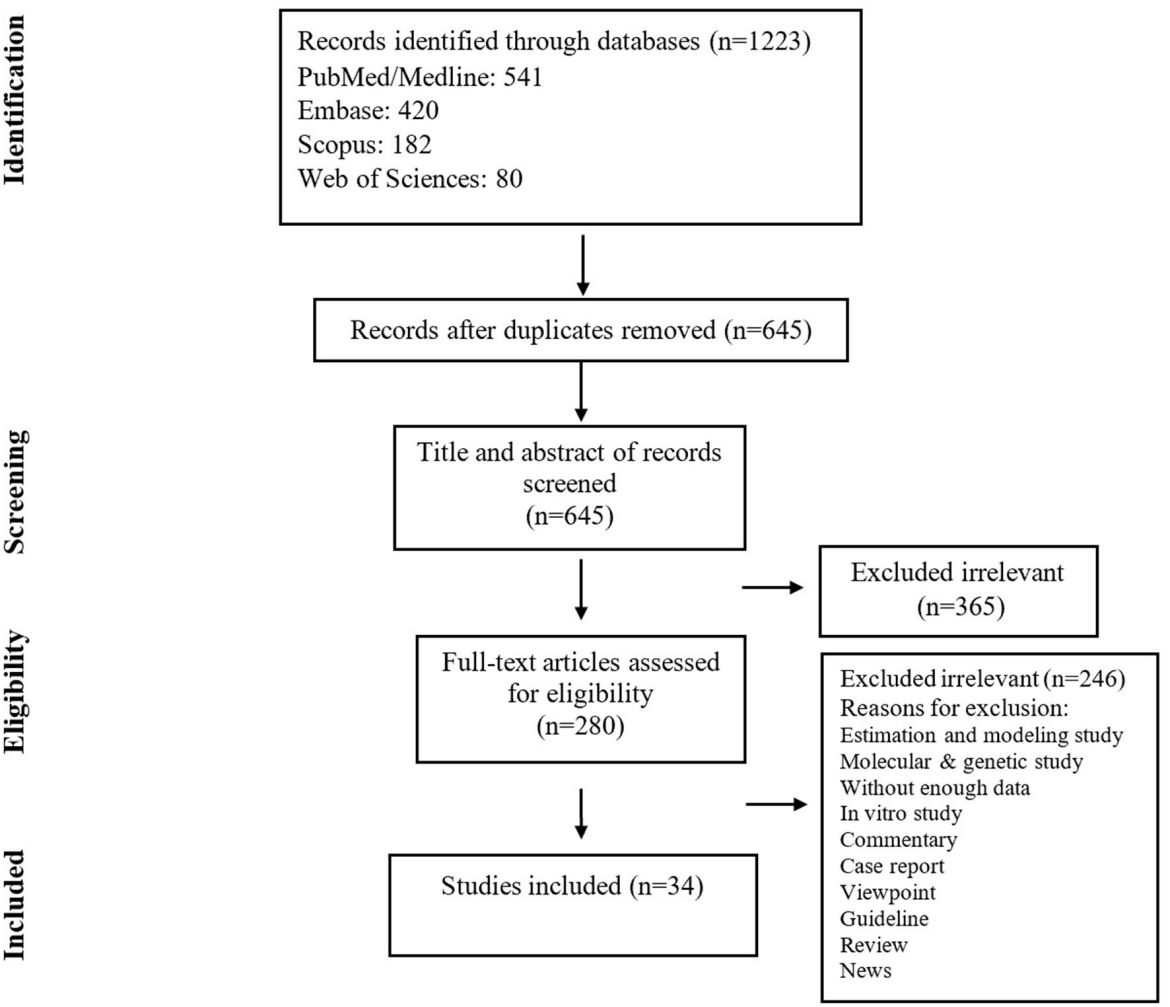
Flow chart of study selection for inclusion in the systematic review and meta-analysis.

The search yielded 1,223 publications, of which 280 potentially eligible studies were identified for full-text review, resulting in 34 studies fulfilling the inclusion criteria (Figure 1) (Table 1). A total of 5,057 patients were included, of which the mean age was 49.0 years. Based on JBI tool, the included studies had a low risk of bias.

**Table 1:** Characteristics of the included studies.

**First author**	**Country**	**Published time**	**Type of study**	**Mean age**	**Male/female**	**Nationality**	**No. of patients**	**Diagnostic methods**
Hui et al. (9)	China	14, Jan, 2020	Case series	NR	NR	Chinese	41	RT-PCR/CT-scan
Xia et al. (10)	China	26, Feb, 2020	Case series	54.5	21M, 9F	Chinese	30	RT-PCR
Xu et al. (11)	China	13, Feb, 2020	Case series	41	M35, F27	Chinese	62	RT-PCR
Zhang et al. (12)	China	7, Feb, 2020	Case series	NR	NR	Chinese	178	RT-PCR
To et al. (13)	China	12, Feb, 2020	Case series	62.5	7M, 5F	Chinese	12	RT-PCR
Zou et al. (14)	China	19, Feb, 2020	Correspondence	59	9M,9F	Chinese	18	RT-PCR
Hoehl et al. (15)	Germany	3, Mar, 2020	Correspondence	35	NR	German	126	RT-PCR/CT-scan
Pan et al. (16)	China	24, Feb, 2020	Correspondence	NR	NR	Chinese	82	RT-PCR/CT-scan
Tang et al. (17)	China	19, Feb, 2020	Cross-sectional	54	98M, 85F	Chinese	183	RT-PCR
Chung et al. (18)	China	4, Feb, 2020	Cross-sectional	51	M13, F8	Chinese	21	RT-PCR/CT-scan
Fang et al. (19)	China	19, Feb, 2020	Cross-sectional	45	29M, 22F	Chinese	51	RT-PCR/CT-scan
Guan et al. (20)	China	28, Feb, 2020	Cross-sectional	47	640M,459F	Chinese	1099	RT-PCR/CT-scan
Huang et al. (21)	China	24, Jan, 2020	Cross-sectional	49	30M,11F	Chinese	41	RT-PCR
Kui et al. (22)	China	7, Feb, 2020	Cross-sectional	57	61M,76F	Chinese	137	RT-PCR
Li et al. (23)	China	29, Jan, 2020	Cross-sectional	52	M238, F187	Chinese	425	RT-PCR/CT-scan
Liu et al. (24)	China	9, Feb, 2020	Cross-sectional	53.6	8M, 4F	Chinese	12	RT-PCR/CT-scan
Wang et al. (25)	China	7, Feb, 2020	Cross-sectional	56	75M, 63F	Chinese	138	RT-PCR/CT-scan
Wu et al. (26)	China	29, Feb, 2020	Cross-sectional	46	39M, 41F	Chinese	80	RT-PCR
Zhang et al. (27)	China	19, Feb, 2020	Cross-sectional	57	71M,69F	Chinese	140	RT-PCR
Ai et al. (28)	China	26, Feb, 2020	Cross-sectional	48.5	M467, F547	Chinese	1014	RT-PCR/CT scan
Pan et al. (29)	China	13, Feb, 2020	Cross-sectional	40	6M, 15F	Chinese	21	RT-PCR/CT-scan
Shi et al. (30)	China	24, Feb, 2020	Cross-sectional	49.5	42M, 39F	Chinese	81	RT-PCR/CT-scan
Yang et al. (31)	China	21, Feb, 2020	Cross-sectional	59.7	35M, 17F	Chinese	52	RT-PCR
Bajema et al. (32)	China	4, Feb, 2020	Cross-sectional	NR	115M, 95F	Chinese	210	RT-PCR/CT-scan
Bernheim et al. (33)	China	20, Feb, 2020	Cross-sectional	45.3	61M, 60F	Chinese	121	RT-PCR
Chen et al. (34)	China	15, Feb, 2020	Cross-sectional	55.5	67M, 32F	Chinese	99	RT-PCR
Pan et al. (35)	China	13, Feb, 2020	Cross-sectional	45	33M, 30F	Chinese	63	RT-PCR
Xu et al. (36)	China	21, Feb, 2020	Cross-sectional	44	29M, 21F	Chinese	50	RT-PCR/CT-scan
Xu et al. (37)	China	28, Feb, 2020	Cross-sectional	50	39M, 51F	Chinese	90	RT-PCR
Chang et al. (38)	China	7, Feb, 2020	Research letter	34	10M, 3F	Chinese	13	RT-PCR/CT-scan
Chen et al. (39)	China	26, Feb, 2020	Research letter	NR	NR	Chinese	85	RT-PCR/CT-scan
Kwok et al. (40)	China	7, Feb, 2020	Research letter	59.8	9M, 5F	Chinese	14	RT-PCR/CT-scan
Hansen et al. (41)	Norway	23 April, 2020	Cross-sectional	72.5	28M,14F	Norwegian	42	RT-PCR/CT-scan
Yu et al. (42)	China	14, May, 2020	Cross-sectional	64	139 M, 87 F	Chinese	226	RT-PCR/CT-scan

### Clinical Manifestations and Comorbidities

The most common signs and symptoms were fever (83.0%, CI 77.5–87.6), cough (65.2%, CI 58.6–71.2), dyspnea (27.4%, CI 19.6–35.2), myalgia/fatigue (34.7%, CI 26.0–44.4), and Sputum production (17.2%, CI 10.8–26.4). Less common symptoms included hemoptysis (2.4%, CI 0.8–6.7), diarrhea (5.7%, CI 3.8–8.6), and nausea/vomiting (5.0%, CI 2.3–10.7) (Table 2).

**Table 2:** Meta-analysis of comorbidities.

	**Pooled frequency**	n/N^*^	**Publication bias**	**Heterogeneity test**	
	**(*p*-value)**		**(*p*-value)**	***I^**2**^* (%)**	***p* value**
Smoking	8.0 (2.3–13.6)	172/1,332	0.06	100	0.00
Hypertension	18.5 (12.7–24.4)	306/1,800	0.98	100	0.00
Cardiovascular disease	14.9 (6.0–23.8)	178/2,031	0.72	100	0.00
Diabetes	10.8 (8.3–13.3)	166/1,932	0.39	100	0.00
Pulmonary disease	3.4 (0.8–6.0)	39/2,031	0.72	100	0.00
Malignancies	2.8 (0.8–4.8)	33/1,816	0.74	100	0.00
Chronic liver disease	8.1 (4.6–11.6)	29/546	0.45	100	0.00
Renal disease	4.4 (0.24–8.6)	17/1,472	0.33	100	0.00

* *n, number of patients with comorbidity; N, total number of patients*.

The most common comorbidities were hypertension (18.5%, CI 12.7–24.4), cardiovascular diseases (14.9%, CI 6.0–23.8), diabetes (10.8%, CI 8.3–13.3), chronic liver disease (8.1, CI 4.6–11.6) and smoking (8.0%, CI 2.3–13.6), respectively (Table 3).

**Table 3:** Meta-analysis of clinical manifestations.

	**Pooled frequency**	n/N^*^	**Publication bias**	**Heterogeneity test**	
	**(95% CI)**		**(*p*-value)**	***I^**2**^* (%)**	***p*-value**
Fever	83.0 (77.5–87.6)	2,073/2,465	0.76	86	0.00
Cough	65.2 (58.6–71.2)	1,689/2,515	0.80	85	0.00
Dyspnea	27.4 (19.6–35.2)	477/2,014	0.42	89	0.00
Myalgia/fatigue	34.7 (26.0–44.4)	742/1,938	0.60	89	0.00
Sputum production	17.2 (10.8–26.4)	480/1,862	0.01	89	0.00
Sore throat	14.5 (10.6–19.5)	224/1,577	0.88	66	0.00
Headache	11.1 (7.7–15.7)	230/1,864	0.30	74	0.00
Diarrhea	5.7 (3.8–8.6)	104/2,041	0.77	66	0.00
Hemoptysis	2.4 (0.8–6.7)	20/1,339	0.77	100	0.00
Anorexia	10.1 (1.0–57.2)	82/1,322	0.73	98	0.00
Nausea/vomiting	5.0 (2.3–10.7)	65/1,563	0.90	85	0.00
Dizziness	8.6 (2.5–26.0)	16/205	0.90	65	0.00
Chest tightness	8.4 (2.5–26.0)	24/256	0.24	78	0.00
Rhinorrhea	9.3 (2.2–31.0)	28/232	0.17	88	0.00
Chills	14.3 (3.0–47.4)	12/111	NA	86	0.00

### Lab Abnormalities and Complications

The most frequent abnormal laboratory findings in patients with COVID-19 were, respectively, elevated C-Reactive Protein (CRP) (72% CI 54.3–84.6), lymphopenia (50.1%, CI 38.0–62.4), elevated Lactate Dehydrogenase (LDH) (41%, CI 22.8–62.0), elevated serum aspartate aminotransferase (19.7%, CI 10.5–33.7), and thrombocytopenia (11.1%, CI 7.7–15.7) (Table 4). Among the confirmed COVID-19 subjects, 14.0% (CI, 6.7–29.0) had viremia. Impaired hepatic function with ALT levels >47.25 U/L was seen in 13.3% (CI 3.2–41.0) of COVID-19 subjects. Acute cardiac injury with troponin levels >28 pg/ml was seen in 12.4% (CI 6.2–23.2). Acute kidney injury was found in 5.5% (CI 1.3–20.8). Shock was reported in 4.0% (CI 1.6–12.0). Finally, 13.0% (CI 4.8–30.0) met the definition of acute respiratory distress syndrome (ARDS).

**Table 4:** Meta-analysis of laboratory findings.

	**Pooled frequency**	n/N^*^	**Publication bias**	**Heterogeneity test**	
	**(95% CI)**		**(*p*-value)**	***I^**2**^* (%)**	***p*-value**
Lymphopenia	50.1 (38.0–62.4)	1,122/1,853	0.08	93	0.00
Lymphocytosis	33.5 (2.4–90.2)	55/93	NA	88	0.00
Neutrophilia	29.7 (19.3–42.7)	60/191	0.51	58.7	0.08
Leukopenia	28.0 (20.0–37.4)	544/1,798	0.89	88	0.00
Leukocytosis	10.8 (5.8–19.1)	165/1,829	0.86	92	0.00
Thrombocytopenia	11.1 (7.7–15.7)	343/1,393	0.00	86	0.00
Anemia	43.5 (30.3–57.7)	79/179	NA	72	0.00
Decreased albumin	51.8 (2.0–98.0)	105/191	0.99	96	0.00
High CRP	72.0 (54.3–84.6)	918/1,681	0.02	96	0.00
High LDH	41.0 (22.8–62.0)	408/1,393	0.32	94	0.00
High ESR	79.7 (66.6–88.5)	143/179	NA	69	0.00
High AST	19.7 (10.5–33.7)	267/1,474	0.70	93	0.00
High ALT	14.6 (7.6–26.3)	191/1,290	0.99	84.8	0.00
High creatinine kinase	14.1 (8.3–23.0)	142/1,453	0.20	84	0.00
High bilirubin	7.9 (2.9–19.0)	95/1,278	0.96	89	0.00
High creatinine	3.3 (1.2–9.1)	20/1,294	0.13	74	0.00
High troponin I	2.4 (0.3–15.0)	1/41	NA	0.00	0.1

### Radiological Characteristics

Chest X-Ray (CXR) and Chest CT scan were the most common imaging modalities used for the diagnosis of COVID-19. The pooled sensitivity of CT scan for detecting COVID-19 was 79.3%. The most common sites of the lung involvement based on chest CT scan were right lower lobe (76.2%, CI 57.8–82.5) followed by the left lower lobe (71.8%, CI 57.8–82.5). Most of the patients (74.8%) had bilateral involvement. The most common pattern of parenchymal involvement was ground-glass opacities (66.0%, CI 51.1–78.0). The Chest CT scan was reported normal in 20.7% of the patients with confirmed RT-PCR results (Table 5).

**Table 5:** Meta-analysis of imaging findings.

**CT Scan**	**Patterns**		**Pooled frequency**	n/N^*^	**Publication bias**	**Heterogeneity test**	
			**(95% CI)**		**(*p*-value)**	***I^**2**^* (%)**	***p*-value**
Location of involvement	Number of affected lobe	Unaffected	20.7 (15.1–27.6)	33/161	0.18	0.0	0.57
		1 lobe	14.8 (7.4–24.0)	52/318	0.22	73	0.00
		2 lobes	9.5 (6.5–12.8)	30/318	0.32	0.0	0.50
		3 lobes	11.7 (7.9–14.6)	36/318	0.64	0.0	0.50
		4 lobes	15.8 (10.3–20.7)	49/318	0.90	40	0.15
		5 lobes	37.2 (32.0–42.3)	118/318	0.50	30	0.22
	Affected lobe (s)	RUL	56.8 (50.6–62.8)	145/255	0.12	52	0.10
		RML	48.6 (42.5–54.8)	124/255	0.07	0.0	0.48
		RLL	76.2 (65.5–84.4)	193/255	0.14	64	0.03
		LUL	56.0 (47.1–64.7)	153/255	0.12	0.0	0.40
		LLL	71.8 (57.8–82.5)	167/234	0.30	76	0.01
	Laterality	Uni lateral	28.8 (16.6–45.2)	62/205	0.80	77	0.01
		Bi lateral	70.6 (55.3–82.5)	142/205	0.20	74	0.01
Pattern of involvement	Pattern of involvement	No involvement	17.2 (11.4–25.0)	193/1,080	0.42	63.0	0.04
		Both of GGO^*^ & consolidation	39.0 (28.1–51.0)	57/142	NA	25	0.24
		GGO without consolidation	66.0 (51.1–78.0)	846/1,365	0.67	90	0.00
		Consolidation without GGO	9.4 (3.3–23.6)	26/274	0.21	82	0.00
	Laterality	Uni lateral	21.8 (12.0–36.3)	101/507	0.63	87	0.00
		Bi lateral	74.8 (62.5–84.0)	405/548	0.29	84	0.00

* *GGO, Ground Glass Opacities*.

### Outcomes

**Figure 2:**
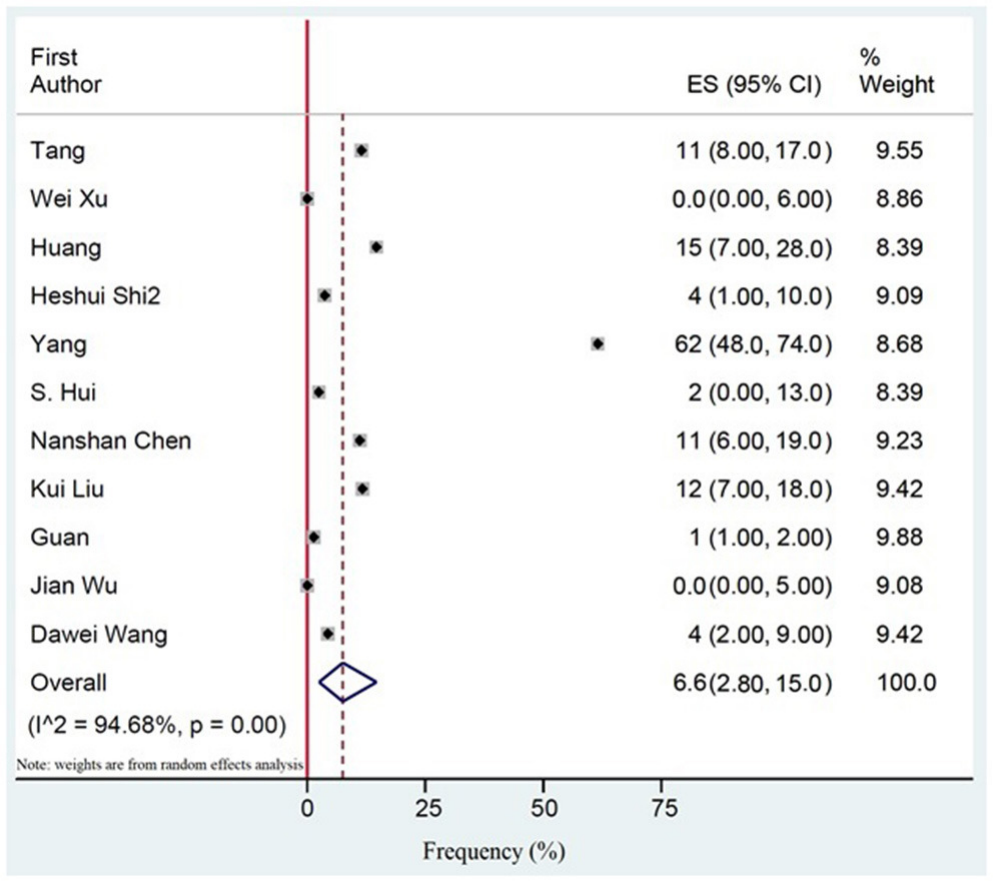
The pooled mortality rate of patients with COVID-19. Effects and summaries were calculated using a random-effects model weighted by study population.

94.6% (CI 73.8–99.1) of the patients with severe COVID-19 were hospitalized. The pooled mortality rate of these patients was 6.6% (CI 2.8–15.0) (Tables 6, 7). Old age, male sex, presence of underlying diseases, higher level of D-dimer, lower level of fibrinogen and anti-thrombin, progressive radiographic deterioration on follow up CT scans, development of ARDS, and requirement of mechanical ventilation were all reported factors associated with increased mortality rate. As shown in Table 8, men had significantly higher mortality in the hospital compared to women (OR 3.4; 95% CI 1.2–9.1, *P* = 0.01). Although ICU admission was higher in men, the difference was not statistically significant. The mean duration between the time of hospitalization and death was 17.5 days with minimum and maximum periods of 14 and 21 days, respectively. The effects and summaries calculated using a random-effects model weighted by the study population is shown in Figure 2.

**Table 6:** Meta-analysis of complications.

	**Pooled frequency**	n/N^*^	**Publication bias**	**Heterogeneity test**	
	**(95% CI)**		**(*p*-value)**	***I^**2**^* (%)**	***p*-value**
RNAemia	14.0 (6.7–29.0)	6/41	NA	0.00	1.00
ARDS	13.0 (4.8–30.0)	142/1,794	0.67	96	0.00
Acute cardiac injury	12.4 (6.2–23.2)	28/243	0.83	65	0.03
Acute kidney injury	5.5 (1.3–20.8)	34/1,441	0.58	93	0.00
Liver failure	13.3 (3.2–41.0)	20/144	0.50	84	0.00
Shock	4.0 (1.6–12.0)	32/1,389	0.60	86	0.00
Hospitalization	94.6 (73.8–99.1)	1,561/1,829	0.76	98	0.00

**Table 7:** Meta-analysis of outcomes.

	**Pooled frequency**	n/N^*^	**Publication bias**	**Heterogeneity test**	
	**(95% CI)**		**(*p*-value)**	***I^**2**^* (%)**	***p*-value**
Discharged	52.7 (36.5–68.4)	486/948	0.44	93	0.00
Death	6.6 (2.8–15.0)	111/2,026	0.50	93	0.00

**Table 8:** Mortality and ICU admission in men vs. women in patients with COVID-19.

	**Pooled OR**	***p*-value**	**Heterogeneity test**	
	**(95% CI)**		***I^**2**^* (%)**	***p*-value**
Mortality in men vs. women	3.4 (1.2–9.1)	0.01	0.00	0.6
ICU admission in men vs. women	1.6 (0.7–3.2)	0.1	0.00	0.5

## Discussion

We evaluated the signs and symptoms, diagnostic modalities, therapeutic measures, and epidemiologic features of COVID-19 to have a better understanding of this pandemic caused by SARS-CoV-2. The pooled mortality rate of these patients was 6.6% overall. We detected several factors that contributed to a worsened outcome including old age, male sex, presence of underlying diseases, and abnormal laboratory finding such as an elevated D-Dimer. Although there was not a significant difference between male and female gender in ICU admissions, male gender showed a significantly higher in-hospital mortality rate.

D-Dimer >1 μg/mL was identified as an associative factor that increased odds of in-hospital death in a study by Zhou et al. (*p* = 0.0033) (43).

Another significant finding in our analysis was the incidence of cardiac injury in 12.4% of the patients, which is a common event seen in a multitude of viral illnesses (44). Gao et al. observed that subjects with influenza (H7N9) and cardiac injury had an elevated risk of mortality (HR = 2.06) (45). In a study by Ludwig et al. which analyzed cardiac biomarkers in influenza patients, 24% of the subjects showed acute cardiac injury ≤30 days after influenza diagnosis and half of the injuries included myocardial infarctions (46). Although our analysis did not show increased mortality risk in patients with cardiac injury, these findings could indicate the potential need for identifying and optimizing cardiac risk factors in COVID-19 patients during the treatment period.

The mean duration between hospitalization and death was 17.5 days (range: 4–21 days), compared to 17.4 days in SARS (47). The overall mortality rate in this study was 6.6%, which is more than twice that was reported earlier (20). Though comparable mortality was reported by Li et al. (7%) and Qian et al. (8.9%) in their meta-analyses, a study by Rodriguez et al. showed a much higher death rate of 13.9% (48–50). On the other hand, a study from the Jiangsu province of China results showed a high cure rate equal to 96.67%. Although the main reason for very low mortality in this study remains unknown, measures including early recognition and centered-quarantine may be contributing factors (51).

Of note, the in-hospital mortality of males was significantly higher than that of females (OR 3.4; 95% CI 1.2–9.1, *P* = 0.01). A similar pattern of higher mortality in males has been reported in previous coronavirus outbreaks of SARS and MERS. Karlberg et al. also reported that the gender-based difference in mortality was higher in younger males (0–44 years) (RR = 2), compared to those of age group 45–74 (RR-1.45) (52). Similarly, the study by Alghamdi et al. showed that the case fatality rate in males was twice that of females in MERS (52 vs. 23%) (6). Although a gender-based difference in the immune response to infections has been suggested as a possible factor, other contributing factors including smoking history and severity of underlying comorbidities cannot be ruled out (53). This is especially of significance in China, where the prevalence of smoking among men (57.6%) is almost 10 times higher that of women (6.7%) (54). This difference in mortality opens the discussion for the need to treat COVID-19 more aggressively in males, including the possibility of earlier intubation and mechanical ventilation in this population. Cigarette Smokers showed to have a higher expression of Angiotensin converting enzyme 2 (ACE2) in lower airways. As it was discussed, ACE2 is the receptor for SARSCoV-2 in the lower respiratory tracts. This finding suggests that smokers are at a higher risk for COVID-19 (55). Therefore we emphasize on smoking cessation especially in the male group with COVID-19. Men smoke more than five times as much as women. (35% in males compared to 6% in females). Although this ratio varies in different countries, it is true that men smoke more in almost all countries (56). These findings can suggest part of the reason behind the significant higher mortality in males with COVID-19. Further investigations are needed to understand this phenomenon.

According to Xiaochen Li et al. male, elder age, leukocytosis, high LDH level, cardiac injury, hyperglycemia and chronic corticosteroid use were related to a higher risk of death in COVID-19. Male group counted for slightly more than half of all their patients (50.9%), however 56.9% of the severe COVID-19 cases were males compared to 45.2% females (*P* = 0.006). They showed that 19.2% of patients with severe COVID-19 were smokers (57).

Ruan et al. studied 68 deceased cases and 82 discharged ones to identify the clinical predictors of COVID-19 mortality, they found a significant difference among patients with Cardiovascular diseases (*p* < 0.001), however, their study didn't show any significant difference in sex ratio between the death group and the discharge group. (*P* < 0.43) (58).

Obesity is a risk factor for comorbid conditions such as cardiovascular diseases which are associated with a higher COVID-19 related deaths. Simonnet et al. showed that invasive mechanical ventilation was significantly associated with male sex (*p* < 0.05) and Body Mass Index (BMI) (*p* < 0.05), independent of age, diabetes, and hypertension (59). Previous studies had shown a low mortality rate in obese and morbid obese patients presenting with ARDS which is defined as obesity paradox. There is still more data required to identify whether this paradox is broken by COVID-19 (60).

According to Zirui Tay et al. there may be alleles on the location of *ACE2* on X-chromosome that confer resistance to COVID-19. This may explain the lower mortality among females. Additionally, estrogen and testosterone sex hormones can modulate the immune response. Therefore, the disease severity may vary based on the hormonal immunoregulation effect (61). In general testosterone have an immunosuppressive effect and estrogen enhances the immunity. Females are less susceptible to viral infections (62).

Recent studies have shown that estrogen upregulates *ACE2* in human atrial myocardium by modulating the local Renin angiotensin aldosterone system (RAAS). Apart from *ACE2, Toll-like receptor (TLR) 7* is also encoded on X-chromosome. TLR7 mediates several immune cell responses (63). Berghöfer et al. showed that *in vitro* exposure of peripheral blood mononuclear cells (PBMCs) to TLR7 ligands results in higher production of interferon-α (IFNα) in cells from females compared to the cells from males (64).

The mechanisms by which androgens such as testosterone decrease the immune response has not been fully understood. Rettew et al. evaluated the acute effect of testosterone through *in vitro* treatment of macrophages generated in absence of androgen. The result was a significant decrease in *TLR4* expression and sensitivity to a TLR4-specific ligand. *In vivo* removal of testosterone resulted in significantly increased TLR4 cell surface expression and higher sensitivity to endotoxin. This may indicate an important mechanism of testosterone immunosuppressive effect (65).

Similar to the sex-based differences in SARAS-CoV2, some studies related to SARS-CoV infection have shown a higher mortality and severity of the disease in males. Karlberg et al. showed a significantly higher case fatality rate in males compared females infected with SARS-CoV (*p* < 0.0001) (52). Channappanavar et al. evaluated the susceptibility to SARS-CoV infection in male mice compared to the age-matched female group. Ovariectomy or estrogen receptor antagonist treatment of female mice showed increased mortality in the SARS-CoV infected mice indicating a protective effect of estrogen receptor signaling (66).

Although around 70% of health and social care workforce worldwide are women and they are in potential exposure to sick patients, most of the studies have shown a higher overall mortality among men with COVID-19. More research is needed to investigate how sex results in different outcomes during the COVID-19 pandemic (63).

This study has several limitations. Due to the rapidly emerging COVID-19 situation around the globe and the novelty of this coronavirus, there is still limited clinical data regarding diagnostic modalities and effective therapeutic measures. Most of the clinical findings were from observational studies. Future clinical trials and animal models are also required to have conclusive clinical information. More studies outside China are needed for comprehensive results that reflect COVID-19 epidemiology globally. Due to the lack of accurate reports of the new cases in different countries, the epidemiologic measures are also limited. As this pandemic is growing fast, future studies are needed for the evaluation of epidemiologic and clinical features of COVID-19.

## Conclusion

COVID-19 has presented with a significant number of mortalities especially among the males around the world. The high rate of hospitalization and case fatality among hospitalized patients along with the lack of intensive care facilities necessitated the identification of the risk factors associated with severe disease and mortality. Males had a significant higher risk of mortality compared to females in our study which was higher than the previous reports from the studies done in China. The reason behind the gender and sex disparity in COVID-19 mortality is still unclear. COVID-19 has been an emerging, rapidly evolving situation. There is still a lot of unknown features of COVID-19 for the broad scientific community to study and identify the risk factors and possible causes of a worse outcome among these patients.

## Future Direction

Further studies are essential on the role of sex hormones on mortality in COVID-19. Moreover, social, lifestyle, and environmental factors should be investigated to understand gender difference in COVID-19 mortality. Studying risk factors associated with mortality can assist us to develop a precise prognostic tool and to personalize treatment in COVID-19.

## Data Availability Statement

All datasets presented in this study are included in the article/supplementary material.

## Author Contributions

MN and MMi: conception and design of study. AT, YF, MA, SHas, PJ, and MN: acquisition of data. MN and MMi: analysis and/or interpretation of data. SHad, MMu, MN, and MMi: drafting and revision of manuscript. All authors contributed to the article and approved the submitted version.

## Conflict of Interest

The authors declare that the research was conducted in the absence of any commercial or financial relationships that could be construed as a potential conflict of interest.
